# The Polynomial Volume Law of Complex Networks in the Context of Local and Global Optimization

**DOI:** 10.1038/s41598-018-29131-0

**Published:** 2018-07-27

**Authors:** Franz-Benjamin Mocnik

**Affiliations:** Institute of Geography, Heidelberg University, Im Neuenheimer Feld 348, 69120 Heidelberg, Germany

## Abstract

Many complex networks expose global hub structures: for some nodes, the number of incident edges far exceeds the average, leading to a small average shortest path length. Such ‘small-world properties’ are often guided by a scale-free power-law distribution of the node degrees, and self-organization inside the network has been identified as a reason driving the emergence of this structure. Small-world networks have recently raised lots of interest, because they capture the global topology of the World-Wide Web, metabolic, and social networks. While small-world networks reflect global structures, little attention is paid to the *local* structure of complex networks. In this article neighbourhoods are demonstrated to share a common local structure in many real complex networks, manifested by a polynomial volume law. This law can, in case of networks that are embedded in space, be explained in terms of the embedding and the properties of Euclidean space. A model of hierarchical spatial networks is introduced to examine the effect of global structures, in particular of hierarchies, on the polynomial volume law. It turns out that the law is robust against the coexistence of such global structures. The local structure of space and global optimization can both be found in transport, brain, and communication networks, which suggests the polynomial volume law, often in combination with hierarchies or other global optimization principles, to be a generic property inherent to many networks.

## Introduction

Networks are used to describe and analyse systems that expose relations between objects. The increasing availability of network datasets – e.g., communication and social networks^1,2^, transport and road networks^3,4^, computer networks, and the World-Wide Web^5^ – has rendered possible a structural comparison of their topological features, which can be shared across different topics and types of network. The distribution *P*(*k*) of the node degree *k* has, e.g., been shown to follow a power law *P*(*k*) ~ *k*^−*γ*^ in many networks, a property that is also called *scale-free*^6,7^: many nodes have a low number of incident edges, while only very few nodes have a high number of these and thus act as ‘hubs’, connecting many different nodes. Shortest paths contain only very few nodes in average, when many hubs are present in a network or the network exposes a strong hierarchy; a network is said to expose *small-world properties*^2,8^ in case of such a short average shortest path length.

Many networks, such as road networks, do not expose such small-world properties. Instead of a short average shortest path length, the average shortest path length of a road network grows much faster for an increasing size of the network; and the node degree is usually limited by 4 or 5. This is despite the fact that road networks are optimized for travel time or travelled distance. Small-world networks, in contrast, expose a low average number of nodes on shortest paths without taking some metric, e.g., travel time or travelled distance, into account. Many different factors constrain the generation of road networks, but there is a major principle that governs their generation: nodes that are close in space are much more often adjacent in the network than distant nodes. This autocorrelation of the road network is also known as *Tobler’s first law of geography* when applied to geographical information in general^9–11^.

The principle of nodes in a neighbourhood being connected by edges has a *local* optimization effect in respect to shortest paths in the network, resembling the realm of Zipf’s principle of least effort^12^. Here, local optimization refers to the fact that the optimization can be executed on smaller neighbourhoods inside the network, while global optimization on the contrary always refers to the entire network. The global effect of hubs has, e.g., extensively been discussed in literature, among others by Watts and Strogatz^2^ and by Barabási and Albert^6^. The following investigates the coexistence of local optimization, caused by the principle of connected neigbourhoods, and global optimization, here exemplified at hierarchies. In particular, I introduce a novel hierarchical spatial network model, which prototypically resembles the coexistence of local spatial structure – the local constellation of edges – and global hierarchies. The volume law^13,14^, which is a result of the local spatial structure, can easily be traced in a network and allows for a reconstruction of the dimension of space. The resulting concept of the dimension of a network is compared to other concepts of fractal dimension. Thereby, the influence of the spatial layout of the network on the evaluation of the dimension is discussed. It is examined at the introduced hierarchical spatial network model in how far local optimization masks the polynomial volume law and impairs the estimation of the dimension. The results are set into context by the analysis of a number of real-world networks, which expose both local and global optimization.

## Results

### Space shaping networks: the polynomial volume law

**Figure 1:**
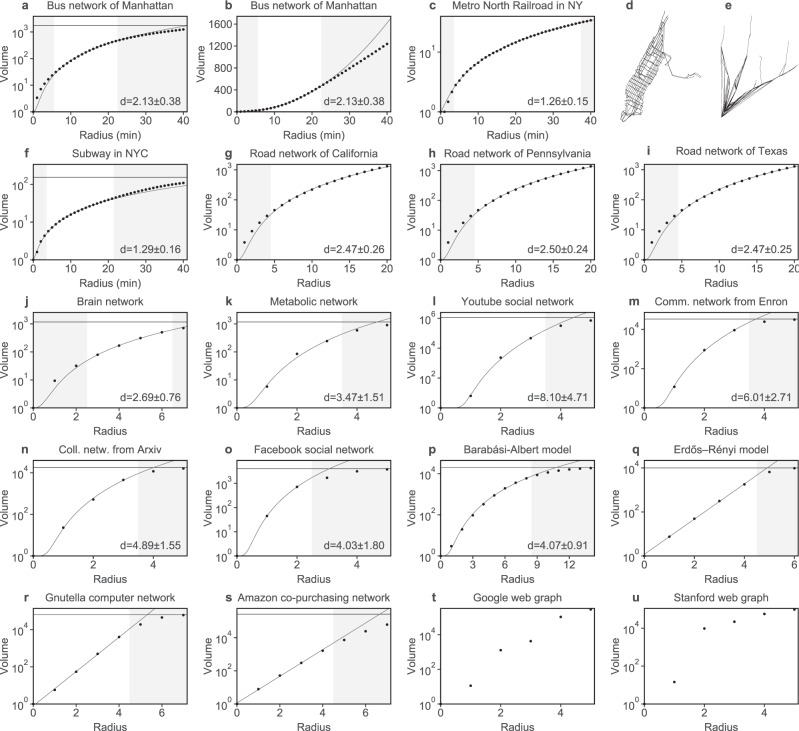
Volumes of real-world networks. Depicted are the arithmetic means of the volumes for 10,000 randomly chosen nodes. The data (dots) in the white part of the plot are fitted by the polynomial volume law (lines) in case of a–p, and by an exponential volume law in case of q–s. The volume is restricted by the total number of nodes, which is represented by a horizontal line. The radius is provided in minutes (**a**–**f**) or by the number of traversed edges (**g**–**u**). In case of polynomial fits, the estimated dimension including the standard deviation is given. (**a**–**c)** Volumes in the bus network of Manhattan, NYC^3^ (with logarithmic and linear scaled axis), and the Metro North Railroad in NY^3^. These transport networks are defined by stops as nodes, and pairs of successive stops as edges with the travel time as weights. (**d, e)** Corresponding transport networks. (**f**) Volumes in the subway network of NYC^3^. (**g**–**i**) Volumes in the road network of California, Pennsylvania, and Texas^4^. (**j**–**o**) Volumes in a brain network^31,40^, the metabolic network of Caenorhabditis elegans^33,34^, the Youtube social network^4^, the email communication network from Enron^4^, the collaboration network of the Arxiv astro physics category^4^, and a network of social circles in Facebook^4^. (**p**) Volumes in a Barabási-Albert model^6^ with 20,000 nodes. (**q**–**t**) Volumes in a Erdős-Rényi model^35,36^ with 10,000 nodes and a probability of 6.495 · 10^−4^, the Gnutella peer-to-peer computer network^4^, and the Amazon product co-purchasing network^4^. The data is fitted by an exponential volume law. (**u**) Volumes in the web graph from a Google programming contest^4^, and the Stanford web graph^4^. The data do neither follow a polynomial nor an exponential volume law.

Networks are in many cases shaped by space: the edges of the network, which represent thematic information, relate in some way to distance in space. If this relation between network and space is strong, both expose similar characteristics. In this case, the network is called to be *spatial*. We can accordingly expect the size of a neighbourhood in the network to depend on its ‘radius’ in a similar way than the volume of a ball does in Euclidean space. Next, we concretize the concept of a neighbourhood in a network, which is called ball in this case. The *ball B*_*n*_(*r*) *of radius r centred at a node n* is defined as the nodes within distance *r* of the node *n*, i.e., as the nodes that can be reached by traversing at most *r* edges or, in a weighted network, as the nodes that can be reached by traversing edges of total weight not more than *r*. The *volume* |*B*_*n*_(*r*)|, in turn, is defined as the number of nodes contained in *B*_*n*_(*r*). Now assume a network that is embedded in an Euclidean space. The volume of a ball in Euclidean space scales as *r*^*d*^, where *r* denotes the radius and *d* the dimension of the embedding space. The volume of a ball in the network can be expected to scale in a similar way, in case of the edges of the network being related to the distance between the nodes. In fact, many real-world networks statistically expose the *polynomial volume law* (Fig. 1a–p):

1 $$|{B}_{n}(r)|=1+k\cdot {r}^{d}$$

where *k* and *d* are some positive real numbers. The left side of the polynomial volume law (Equation 1) refers to the volume in the network, while the right side refers to the volume of a ball in Euclidean space incremented by 1, reflecting that the ball of radius 0 in the network contains exactly one node. This law has been discussed previously by Song *et al*.^13^ and Shanker^14^ but has to my knowledge never been examined in detail with respect to hierarchies inside the network.

When statistically fitting the volume of a ball for different radii *r* in a network by Equation 1, the parameter *d* can, in contrast to Euclidean space, be a non-integer. This is in particular the case if the network does not equally ‘extend’ to every dimension of the embedding space. While a meaningful embedding in space is able to *explain* why many networks expose the polynomial volume law, such an embedding is not needed to compute the volumes |*B*_*n*_(*r*)| for different nodes *n* and different radii *r*, and, in turn, for determining whether a network follows the law and which real number *d* – called the dimension of the network – fits best.

### Comparison to the fractal dimension

The dimension derived by the polynomial volume law is in many aspects similar to other approaches that relate a network to the dimension of the space it is embedded in. Most notable, the *box counting dimension*, also called *Minkowski*-*Bouligand dimension* or *fractal dimension*, establishes a relation between the embedding space and the network by comparing the complexity at different scales^13,15^. Thereby, space is tessellated with a grid of boxes and the number of boxes containing at least one node (or alternatively, the number of boxes intersecting at least one edge of the network) is determined. As a result, one is able to conclude the dimension by the relation between the number of such boxes and their side lengths. The box counting dimension has been discussed in various articles, among others, in respect to self similarity in networks^16–18^. Efficient algorithms for the computation of this dimension have been published^19^. A comparison of such algorithms has been provided by Song *et al*.^20^. Even the idea of the box counting dimension has been subject to advancements^17,21^. The box counting dimension has been discussed in various contexts, among others, in the geographical context^22,23^.

Approaches similar to the dimension defined by the polynomial volume law have been discussed in literature. For instance, Daqing *et al*.^24^ have considered the average Euclidean distance in space *E*_*n*_(*r*) from a centre node *n* to all nodes inside a ball *B*_*n*_(*r*), i.e., to all nodes that can be reached by traversing at most *r* edges of the network. This average Euclidean distance has been compared to the volume of the ball in the network, as defined previously^24^. Thereby, a number referred to as the dimension is assigned to the network, much similar as in case of the polynomial volume law. The comparison of the volume of a ball *B*_*n*_(*r*) to the average distance *E*_*n*_(*r*) instead as to 1 + *k* · *r*^*d*^ has two major consequences. First, the average distance *E*_*n*_(*r*) explicitly includes the concept of Euclidean distance, which presumes the network to be explicitly embedded in an Euclidean space. The comparison to 1 + *k* · *r*^*d*^ can though also be performed for an abstract network, without any knowledge about the potential location of a node. Secondly, the comparison of the volume of a ball *B*_*n*_(*r*) to the average distance *E*_*n*_(*r*) examines how topological and Euclidean aspects of the very network relate, while the comparison to 1 + *k* · *r*^*d*^ how the topological aspects of the network relate to the universal polynomial law that describes the Euclidean volume of a ball in general. In short, the considerations of Daqing *et al*.^24^ include an explicit Euclidean embedding of the network, while the polynomial volume law 1 + *k* · *r*^*d*^ only compares to Euclidean spaces in general.

Further approaches exist to characterize networks by their dimension. Daqing *et al*.^24^ examine the root mean square displacement by a random walk. Song *et al*.^13^ have pointed out that the different estimations of the dimension of space do not coincide in some cases, e.g., in case of small-world networks.

**Figure 2:**
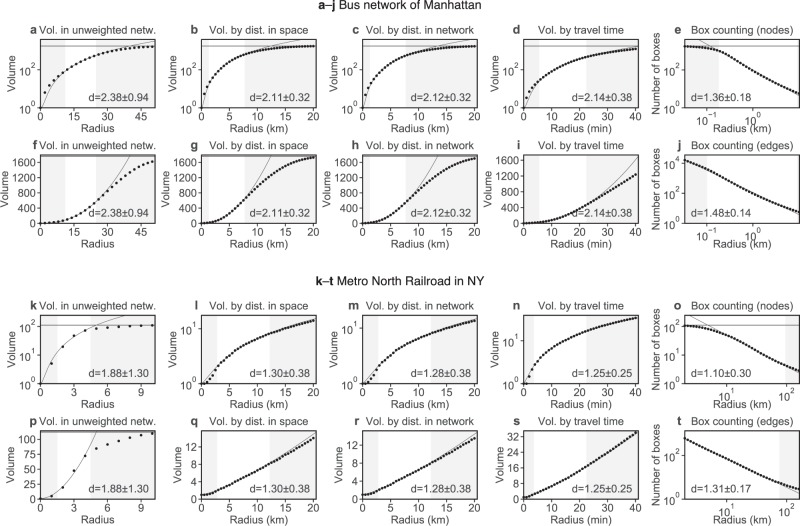
Different concepts of network dimension. (**a**–**d**, **f**–**i**, **k**–**n**, **p**–**s**) Estimation of the dimension by the volume in the unweighted network, or in the weighted network considering distance in space, distance in network, or travel time respectively. For each computation of a dimension, the arithmetic means of the volumes for 10,000 randomly chosen nodes have been examined. The data (dots) in the white part of the plot are fitted by the polynomial volume law (lines). The volume is restricted by the total number of nodes, which is represented by a horizontal line. The estimated dimension includes information about the standard deviation. (**e, j, o, t**) Estimations of the dimension by the box counting method. For each computation of a dimension, the average of 500 grids of boxes randomly translated in space has been examined. The data (dots) in the white part of the plot are fitted by a double logarithmic law (lines). The boxes are, in case of (**e, o**) restricted by the total number of nodes, which is represented by a horizontal line. The estimated dimension includes information about the standard deviation.

Figure 2 compares different types of network dimensions for two real-world networks, the Bus network of Manhattan and the Metro North Railroad in NY. The figure shows the dimensions resulting from the polynomial volume law in four variants. First, the volumes by the distance in the network are determined by the distance in an unweighted network. Secondly, the distance in Euclidean space between two adjacent nodes is used as weight, and the volumes are computed for the weighted network. Thirdly, the distances between adjacent nodes is computed in the embedded network, i.e., the weights correspond to the distance a bus or train needs to travel. Fourthly, travel times are used as weights. In addition to these dimensions resulting from the polynomial volume law, the box counting dimension is computed by counting the boxes that contain a node of the network, or by counting the boxes that intersect an edge of the network.

The different concepts of dimension result in different values, as can be seen in Fig. 2. The estimated dimensions by the volumes in the weighted network are very similar, both in the example of the bus network as well as of the railroad network. In the unweighted network however, the estimation of the dimension is higher and is subject to a large standard deviation. In the case of the Metro North Railroad, a fit can hardly be made because the effective diameter of the network is small, which is reflected by the small range of the fit. The box counting dimension provides lower estimations when referring to nodes compared to when referring to edges. Both, variants of the box counting dimension provide lower estimates than the polynomial volume law, which is much likely an artefact of the dimensions to reflect different concepts: the box counting dimension compares complexity at different scales while the polynomial volume law carries over concepts from Euclidean space to the network. Despite of this difference, the box counting dimension is higher in case of the Bus network compared to the Metro North Railroad, which is consistent with the polynomial volume law.

### Local and global optimization principles

Many generation principles are known to guide the emergence of networks. Among them are principles that avoid edges between distant nodes in space, leading to a large diameter of the network, as well as principles that minimize the average distance between the nodes of the network and thus lead to small-world networks. In the following, we discuss factors that lead to these principles and how they relate.

**Figure 3:**
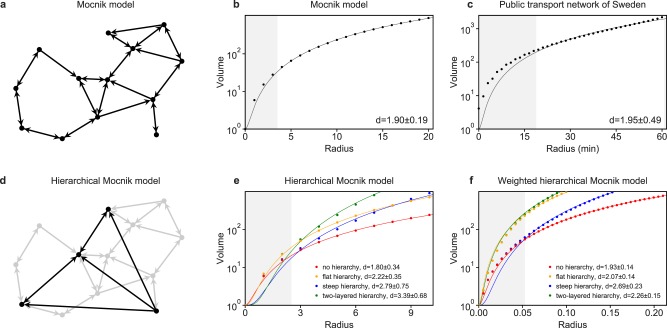
Hierarchical spatial networks. In (**b**–**c**) and (**e**–**f**) the arithmetic means of the volumes for 10,000 arbitrary chosen nodes are depicted. The estimated dimension including the standard deviation is given. (**a**) Mocnik model with 13 nodes and *ρ* = 1.5. (**b**) Volumes of the undirected network associated to a Mocnik model with 10,000 nodes in two-dimensional space and *ρ* = 1.8. (**c**) Volumes in the public transport network of Sweden^41^, which is a multi-modal and hierarchical network. The data is fitted by the polynomial volume law. (**d**) Hierarchical Mocnik model, with the base layer depicted in grey and layer 1, in black. (**e**) Volumes of the undirected network associated to two-dimensional hierarchical Mocnik models with *ρ* = 1.8. (In fact, the value of *ρ* is below 2 for most real-world networks.) The following hierarchies are depicted: no hierarchy (*N*_0_ = 10000), flat hierarchy (*N*_0_ = 10000, *N*_1_ = 1000), steep hierarchy (*N*_0_ = 10000, *N*_1_ = 100), and two-layered hierarchy (*N*_0_ = 10000, *N*_1_ = 1000, *N*_2_ = 100). (**f**) Volumes of the undirected network associated to two-dimensional weighted hierarchical Mocnik models with *ρ* = 1.8. The following hierarchies are depicted: no hierarchy (*N*_0_ = 10000; *w*_0_ = 1), flat hierarchy (*N*_0_ = 10000, *N*_1_ = 3000; *w*_0_ = 1, *w*_1_ = 0.375), steep hierarchy (*N*_0_ = 10000, *N*_1_ = 100; *w*_0_ = 1, *w*_1_ = 0.25), and two-layered hierarchy (*N*_0_ = 10000, *N*_1_ = 3000, *N*_2_ = 100; *w*_0_ = 1, *w*_1_ = 0.375, *w*_2_ = 0.25).

The polynomial volume law is often the result of a *local optimization principle*: assuming that the costs of an edge depend on its length, how can a node be adjacent to as many nodes of the network as possible? This principle is of local nature because it can be answered independently for each node. In the resulting network, a node is obviously adjacent to the nodes of its neighbourhood in space while being non-adjacent to more distant nodes. This local optimization principle has been resembled by different models. An approach is to introduce edges with a probability that depends on the distance of the nodes in space, e.g., with a probability of *P*(*l*) = *α* exp(−*l*/*l*_0_) with positive values *α* and *l*_0_^25^, or with a probability of *P*(*l*) = 1 if *l* < *l*_0_ and *P*(*l*) = 0 otherwise^26^. Another model, which we refer to as the *spatial network model* or *Mocnik model*, has been proposed by Mocnik^27,28^. Assume a number of nodes being embedded in space. We then introduce a directed edge (*n*_1_, *n*_2_) if and only if

2 $${\rm{dist}}({n}_{1},{n}_{2})\le \rho \cdot \mathop{{\rm{\min }}}\limits_{m\ne {n}_{1}}\,{\rm{dist}}({n}_{1},m)$$

where dist denotes the Euclidean distance and *ρ* > 1 a parameter that influences the density of the network (Fig. 3a). The model prototypically resembles Tobler’s first law of geography: ‘everything is related to everything else, but near things are more related than distant things’^9–11^. Despite this, the model applies to other scales than the geographical scale as well.

The Mocnik model follows the polynomial volume law. When the nodes are randomly distributed in space with a uniform distribution, the edges introduced by the model reflect properties of space, e.g., the existence of proximity. As a consequence, the number of edges is expected to be linear in the number of nodes^27^; and the dimension of space has an impact on the configuration of the edges. In fact, the volume of the undirected network associated to this model follows the polynomial volume law (Fig. 3b), in which the exponent *d* resembles the dimension of space. The parameter *ρ* determines the density of the network, i.e., the ratio of the number of actual edges to the maximal number of edges in a simple network. Thereby, *ρ* has an impact on the configuration of edges as well, but in a model with an infinite number of nodes *ρ* does not influence the exponent *d* when fitting by the polynomial volume law. Even in middle and large size networks, the influence of *ρ* does practically not mask the impact of the dimension^28^. The Mocnik model – a network embedded in space with only short-distance edges – can thus serve as an explanation of the polynomial volume law by local optimization.

In contrast to the local optimization principle that maximizes the number of adjacent nodes, *global optimization principles* often play a major role: assuming that a network shall only contain a limited number of edges, how can the average distance between pairs of nodes statistically be minimized for the entire network? This principle does not refer to some nodes only rather than to the entire network. If a network complies with this optimization principle, most shortest paths between two randomly chosen nodes are, in fact, very short, but single nodes may suffer from a longer distance to large parts of the network. Among the models that create such small-world networks are the Watts-Strogatz model^2^ and the Barabási-Albert model^6^.

Real-world networks are often organized by both local and global optimization principles. Local optimization principles naturally occur when the costs of an edge positively correlate to its length, which is the case for physical networks (road and railway networks, etc.) but also for many types of communication networks (network of postal delivery services, the telephone network, etc.). Global optimization, in contrast, often minimizes the average length of shortest paths by introducing edges between distant nodes in space. Such global principles naturally occur for networks that are, at least in large parts, of virtual nature, e.g., to friend networks in social media. Most networks are though guided by a combination of local and global optimization to achieve a balance between costs and the length of shortest paths in the network. In the remainder of the article, we explore the interaction between local and global optimization and discuss its effect on the polynomial volume law.

### A model of hierarchical spatial networks

The Mocnik model^27^ is guided by a local optimization principle, as becomes apparent by Equation 2. In order to study the interaction between local and global optimization principles, we extend the Mocnik model in the following to a *hierarchical Mocnik model*. Thereby, the hierarchical model aims at including a global optimization principle by introducing different layers in the network. This hierarchy is, as we show later, to some extent compatible with local optimization principles. If the layers of the hierarchy share nodes, i.e., if they are connected, shortest paths in the network become shorter in comparison to the non-hierarchical model, because shortest paths often traverse higher layers of the hierarchy, which are more efficient in bridging space.

Hierarchies and the principle of layered networks can be found in many transport networks. For instance, many road networks expose layers: motorways, primary, secondary and tertiary roads, residential roads, etc. Railway networks often consist of long-distance and of local trains, the former which usually have less stops and are much faster than the latter. The shortest route in a railway network incorporates thus often a local train to a larger station, then long-distance trains, and potentially another local train. The universal nature of this principle has been widely recognized, and important routing algorithms take thus advantage of hierarchies inside the data^29^.

The hierarchical Mocnik model makes use of the non-hierarchical model in every layer of the hierarchy. Assume a number of node sets ${N}_{l}\subset {N}_{l-1}\subset \ldots \subset {N}_{0}$ to be embedded in space, which each correspond to one layer of the network. Then, for each layer consisting of nodes *N*_*i*_, edges *E*_*i*_ are created in accordance to Equation 2. The nodes *N*_*i*_ of a layer together with the corresponding edges *E*_*i*_ are, accordingly, a Mocnik model. The lowest layer *N*_0_ will in the following be referred to as the *base layer* of the network.

Local and global optimization coexist in the hierarchical Mocnik model. The base layer is guided by local optimization in the same way as the non-hierarchical Mocnik model: in each neighbourhood, the constellation of edges is optimized for a high number of adjacent nodes. In addition to the non-hierarchical model, the layers of the hierarchical variant expose different degrees of local and global optimization. The less nodes a layer contains, i.e., the higher the layer in the hierarchy, the more global the optimization becomes. The optimization in a higher layer of the hierarchy only involves some nodes of the network while ignoring many other ones, which means that the optimization is not any longer performed in spatial neighbourhoods.

Even a weighted variant of the hierarchical Mocnik model can be introduced, in which the edges are complemented by weights. Thereby, the weight of an edge corresponds to its lengths in Euclidean space. The introduction of a new layer to an existing Mocnik model makes shortest paths potentially shorter even in case of the weighted model. While the nodes stay untouched, new edges are introduced in each layer of the network but none is removed. Accordingly, some nodes are directly connected in some layer *E*_*i*_ while the shortest path in *E*_*j*_ with *j* < *i* is potentially longer – triangle inequality applies.

The weights of the weighted variant of the hierarchical Mocnik model can even be systematically adjusted in respect to the hierarchy. The weights *w*_*i*_ for each layer of the hierarchy reflect that the layers are of different speed, require different communication costs, etc. In the *weighted hierarchical Mocnik model*, the weight of an edge in layer *i* is defined as the length of the edge in Euclidean space, multiplied by *w*_*i*_. If all *w*_*i*_ are equal to 1, the weights are, accordingly, equal to the length of the edges. The resulting network usually consists of many edges with low weights in the base layer, and only some edges with slightly greater weights in higher layers (Fig. 3d). Such a weighted hierarchical model is very similar to transport networks, in which local transport connects adjacent places, and more distant places are connected by motorways or long-distance trains operating at a higher speed.

### Synthesis of local and global optimization principles

The hierarchical Mocnik model is characterized by local optimization in each layer and global optimization by hierarchies. In case of one layer only, the model is prototypically characterized by local optimization. In case of several layers there exist shortcuts, which expose characteristics of global optimization and lead to small-world properties in the network. Here, we examine the impact of coexisting local and global optimization principles on the polynomial volume law.

The volume of a non-weighted hierarchical network is always larger than the volume in the base layer of a network. In fact, the volume increases when a layer with 3000 nodes (flat hierarchy) is introduced, or when a layer with 100 nodes (steep hierarchy) is introduced on top of the base layer in the example of Fig. 3e. For smaller radii, the volume is larger for flatter hierarchies, because more nodes are adjacent to the higher layer and shortest paths between two nodes of the same small spatial neighbourhood more often traverse nodes of a higher layer. At larger radii, the increase in volume is, though, larger for steeper hierarchies, because the shortcuts introduced by the hierarchy are more efficient. If several layers are added on top of each other, the increase of volume at smaller radii is guided by the lower layers of the hierarchy, and the increase of volume at larger radii is guided by the higher layers of the hierarchy.

While the influence of the hierarchies are obvious in case that the base layer of the network is known, such comparisons can hardy be drawn in general. Instead, we may ask how the hierarchies affect the measured volumes in comparison to the fit (to the polynomial volume law), because the difference between the fit and the actual data can be examined without any knowledge about prevailing layers. In fact, the fit underestimates the volume and overestimates the exponent *d*, the dimension, in different ways. For a steep hierarchy with much less nodes in a higher layer than in the base layer, the fit underestimates the volume at smaller radii (Fig. 3e). This effect is independent of whether there exist additional layers in the hierarchy in case of a non-weighted model, i e., the number of nodes in the highest layer of the hierarchy has a major impact on the underestimation. At the same time, the rate of growth is higher in case of a steep hierarchy for larger radii, leading to higher estimates of the dimension. If the hierarchy is flatter, the estimated dimension is lower than in case of a steep hierarchy but higher than for the base layer alone.

The underestimation of the volume and overestimation of the dimension can also be observed in case of the weighted hierarchical Mocnik model (Fig. 3f). The effect is though less significant because the lengths of the edges is taken into account, and higher layers provide less effective shortcuts than in the non-weighted model. The presence of a layer with more nodes can even obfuscate the effect of a layer with much less nodes in case of the weighted hierarchical Mocnik model. The fact that both kinds of hierarchical Mocnik models follow a polynomial volume law, despite being layered networks with several hierarchies, suggests that the polynomial power law is robust and not necessarily masked by other structures inside the network.

## Discussion

The examination of the Mocnik model has demonstrated that global optimization leads to an underestimation of the volume at lower radii and an overestimation of the dimension when fitting to the polynomial volume law. Despite having only examined hierarchical structures as an example of global optimization, the underestimation of the volume and the overestimation of the dimension origins from the existence of shortcuts in the network, which suggests that other global optimization principles have similar effects. Here we study real-world networks in order to trace the effects of coexisting local and global optimization at real examples.

### Examples of spatial networks

The considered transport networks follow a polynomial volume law with an exponent *d* between 1.2 and 2.5 (Figs 1a–i and 3c). The bus network of Manhattan, e.g., is highly-branched, the exponent *d* = 2.13 ± 0.38 reflecting that the network exhausts the two-dimensional space of Manhattan (Fig 1a, b, d). The Metro North Railroad in NY is, in contrast, a lowly-branched railway line with exponent *d* = 1.26 ± 0.15, which reflects that it does not exhaust space (Fig. 1c, e). The fact that the estimation of the dimension is biased towards a larger number than 2 in case of the bus network of Manhattan can be explained by the hierarchies inherent to the network. When the radius approaches the travel time from the west to the east of Manhattan (≈20 min), the volume increases more linearly due to the elongated shape of Manhattan (Fig. 1a, b, d). The road networks of California, Pennsylvania, and Texas follow the polynomial volume law as well, with exponent *d* = 2.47 ± 0.26, *d* = 2.50 ± 0.24, and *d* = 2.47 ± 0.25 respectively (Fig. 1g–i). These road networks are, in fact, strongly hierarchically, which is why the estimated dimension is higher than 2 and the volume is underestimated for lower radii. The same applies to the public transport network of Sweden (Fig. 3c), which incorporates many modes of transport with different service areas and travel speeds.

The polynomial law also applies to examples of non-geographical networks. Human connectomes^30^, which describe neural connections in the brain, can be represented as networks^31,32^ with small brain regions, containing a collection of neurones, as nodes; and fibres between these regions, as edges. An exemplary brain network follows the polynomial volume law with exponent *d* = 2.69 ± 0.76 (Fig. 1j). A reason for why the human connectome exposes this law is, at least in parts, its existence in three-dimensional space. In addition, the folding of the more two-dimensional cerebral cortex, the ‘outer shell’ of the brain, might be a reason for the dimension of the fit to be smaller than 3.

### Other networks following the polynomial volume law

Some networks follow the polynomial volume law despite having no obvious embedding into space. Among them are the metabolic network of Caenorhabditis elegans^33,34^, a social friendship-network, an email communication network, a collaboration network, and the Barabási-Albert model^6^, but often with a much higher exponent (Fig. 1k–p). The volume can though hardly be examined in many of these and other small-world networks because their diameter is low by definition – global optimization principles play a role. Hence saturation effects limit the increase of volume when the volume is computed for increasing radii. The characterization of the structure of balls in a network by their volume is thus only meaningful if the network provides some notion of locality.

### Networks guided by other principles

Many networks expose a power law, often indicating a local organization principle. In fact, such a law is prototypically met if all nodes have the same node degree. The volume in a tree with constant branching factor *b*, e.g., relates to the radius *r* essentially by the *power law b*^*r*^. Balls in such networks are organized differently compared to those of spatial networks, and they can thus often not naturally be embedded in space. Volumes in the Erdős-Rényi model^35,36^, in case of a low number of edges, follow an *exponential law* (Fig. 1q). Examples of a peer-to-peer computer network and a product co-purchasing network follow an exponential law as well (Fig. 1r, s). There exist though networks that follow neither a polynomial nor a power or exponential law, e.g., two web graphs (Fig. 1t, u).

## Conclusion and Future Work

Space shapes networks, a fact that manifests itself in a polynomial volume law. This law has been demonstrated for several networks, and many other networks that are naturally embedded in space can be expected to expose this law as well. The characterization of networks by the volume of balls has been shown to reveal generic design principles, e.g., a local optimization in regards to short average shortest path lengths in the network. Such local optimization is often accompanied by global optimization principles that create shortcuts in the network and often lead to a much smaller diameter of the network. As an example, such global optimization is inherent to hierarchical networks. We have extended the Mocnik model to incorporate hierarchies. The resulting network prototypically demonstrates the influence of global optimization on spatial networks: the effect of local optimization becomes visible at smaller radii (polynomial volume law) and global optimization at the existence or non-existence of larger radii (small diameter of the network). If the volumes are fitted, the volume is underestimated for low radii, and the dimension is overestimated. Besides this effect, global optimization principles do, in many cases, not destroy the polynomial volume law in its core. Local and global optimization principles, here studied at the example of hierarchies, thus complement each other. Such observations can not only be made for the hierarchical network model but also for many real-world networks.

The exploration of how local and global organization principles and their mechanisms relate allows for advancements in the understanding of geographical, brain, social, and other complex networks, and it may decipher some of the principles that guide the emergence of such networks. As an example of such advancements, algorithms can be optimized to take advantage of the fact that many networks expose a polynomial volume law at least heuristically. Dijkstra’s algorithm^37^, used for graph traversal, does not take advantage of the embedding of a network in Euclidean space. This is in contrast to the A* algorithm^38^, which takes, as an extension of Dijkstra’s algorithm, advantage of such an embedding when choosing the Euclidean distance as heuristic cost function. It is subject to further investigations to examine how such improvements can be made for other algorithms. As soon as the polynomial volume law is met in a network, it exposes some notion of locality, similar to a network embedded in Euclidean space. This allows network-related algorithms to take advantage of locality, at least heuristically, and thus to be improved in terms of efficiency.

The introduced hierarchical Mocnik model can serve as a general model of objects and relations in between, in case that both can or are naturally embedded in space, i.e., of spatial data or information. The model does though not reflect all characteristics of real examples. Further research might tailor the model to reflect even more accurately the characteristics of road networks, brain networks, or communications in space. Thereby, the characteristics of these networks need to be formally understood and translated to the Mocnik model. In particular, a better understanding is needed of how the number of layers and their size can be estimated in a real-world network, which allows for a more realistic modelling. It might even be explored how the choice of the layers of the hierarchical Mocnik model can be optimized. Many spatial networks even evolve over time. Principles that guide the evolution of a network can, in many cases, be used to understand the emergence of a network, e.g., in case of the Barabási-Albert model. Further investigations of the Mocnik model might reveal similar principles that generate the model iteratively and explain its properties in terms of its evolution.

## Methods

The transport networks considered in this article consist of stops and stations as nodes, and pairs of successive stops of the same trip as edges. The weights of the edges refer to the travel time, where the start point in time of the travel is defined as the arithmetic mean of the preceding arrival and the departure of the travel represented by the edge, and the end point as the mean of the arrival and the subsequent departure. The public transport network of Sweden (Fig. 3c) contains data from almost all public transport providers in Sweden. The brain network (Fig. 1j) refers to the network GROUP_MATRIX_HD_gr2 of the referenced dataset. All other datasets are adopted from the respective references provided in the captions.

**Figure 4:**
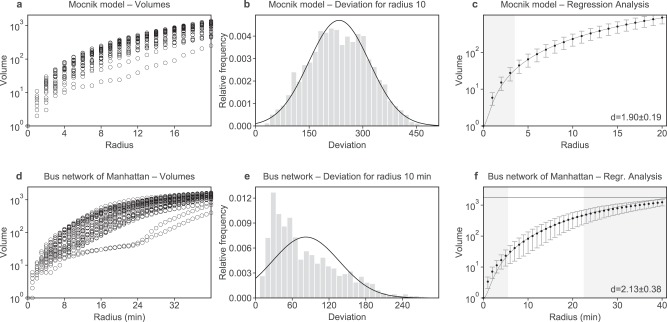
Methods: Statistics. (**a**, **d**) Volumes for 30 randomly chosen nodes. (**b, e**) The deviation of the volumes for radius 10 in the Mocnik model roughly follows a normal distribution. The deviation of the volumes for radius 10 min in the Bus network of Manhattan differs from a normal distribution. (**c, f**) Regression analysis using the method of least squares.

The volume is computed for 10,000 randomly chosen nodes of the largest connected component of each network, or of the associated undirected network in case of a directed network (Fig. 4a, d). The deviation of the volume roughly follows a normal distribution (Fig. 4b). A regression analysis is performed by the method of least squares for the arithmetic means, in consideration of the standard deviations computed for each radius (Fig. 4c, f). The regression analysis excludes very small and larger radii for some networks, because the computed volumes for these radii sometimes differ from the theoretic expectations: First, the finiteness of the network sets an upper limit to the volume, leading to lower volumes for balls near the boundary of the network. Secondly, volumes in a network are discrete and differ thus for small radii from the volumes in Euclidean space.

The box counting method is applied to 500 grids of boxes. These boxes have been randomly translated in space to minimize effects of the tessellation of space. For determining the boxes that intersect an edge, the edges have been discretized by points (25 points per side length of the smallest examined box).

### Data availability

All sources of data have been cited. An implementation of the described Mocnik model is published as part of NetworKit^39^ (https://github.com/kit-parco/networkit), an open-source toolkit for large-scale network analysis.

## References

[1] Newman MEJ (2001). The structure of scientific collaboration networks. Proc. Natl. Acad. Set United States Am..

[2] Watts DJ, Strogatz SH (1998). Collective dynamics of small-world networks. Nat..

[3] Metropolitan Transportation Authority. MTA data, http://web.mta.info/developers/developer-data-terms.html - accessed on April 14, 2016 (2016).

[4] Leskovec, J. & Krevl, A. Stanford large network dataset collection. http://snap.stanford.edu/data - accessed on June 14, 2018 (2018).

[5] Albert R, Jeong H, Barabási A-L (1999). Diameter of the World-Wide Web. Nat..

[6] Barabási A-L, Albert R (1999). Emergence of scaling in random networks. Sci..

[7] Barabási A-L, Bonabeau E (2003). Scale-free networks. Sci. Am..

[8] Barthélémy M, Amaral LAN (1999). Small-world networks: Evidence for a crossover picture. Phys. Rev. Lett..

[9] Tobler WR (1970). A computer movie simulating urban growth in the Detroit region. Econ. Geogr..

[10] Tobler WR (2004). On the first law of geography: A reply. Annals Assoc. Am. Geogr..

[11] Miller HJ (2004). Tobler’s first law and spatial analysis. Annals Assoc. Am. Geogr..

[12] Zipf GK (1947). The hypothesis of the minimum equation as a unifying social principle: with attempted synthesis. Am. Sociol. Rev..

[13] Song C, Havlin S, Makse HA (2005). Self-similarity of complex networks. Nat..

[14] Shanker O (2007). Defining dimension of a complex network. Mod. Phys. Lett. B.

[15] Mandelbrot, B. B. *The fractal geometry of nature* (Freeman, New York, 1982).

[16] Gallos LK, Song C, Makse HA (2007). A review of fractality and self-similarity in complex networks. Phys. A.

[17] Zhou W-X, Jiang Z-Q, Sornette D (2007). Exploring self-similarity of complex cellular networks: The edge-covering method with simulated annealing and log-periodic sampling. Phys. A.

[18] Li, B.-G., Yu, Z.-G. & Zhou, Y. Fractal and multifractal properties of a family of fractal networks. *J. Stat. Mech. Theory Exp*. 2014 (2014).

[19] Schneider CM, Kesselring TA, Andrade JS, Herrmann HJ (2012). Box-covering algorithm for fractal dimension of complex networks. Phys. Rev. E.

[20] Song, C., Gallos, L. K., Havlin, S. & Makse, H. A. How to calculate the fractal dimension of a complex network: the box covering algorithm. *J. Stat. Mech. Theory Exp*. **2007** (2007).

[21] Wei, D.-J. *et al*. Box-covering algorithm for fractal dimension of weighted networks. *Sci. Reports* **3** (2013). 10.1038/srep03049 PMC6505717 24157896

[22] Li, R. *et al*. Simple spatial scaling rules behind complex cities. *Nat. Commun*. **8** (2017). 10.1038/s41467-017-01882-w PMC5705765 29184073

[23] Batty, M. & Longley, P. A. *Fractal cities. A geometry of form and function* (Academic Press, London, 1994).

[24] Daqing L, Kosmidis K, Bunde A, Havlin S (2001). Dimension of spatially embedded networks. Nat. Phys..

[25] Waxman BM (1988). Routing of multipoint connections. IEEE J. on Sel. Areas Commun..

[26] Huson ML, Sen A (1995). Broadcast scheduling algorithms for radio networks. Proc. Mil. Commun. Conf. (MILCOM).

[27] Mocnik, F.-B. & Frank, A. U. Modelling spatial structures. *Proc. 12th Conf. on Spatial Inf. Theory (COSIT)*, 44–64 (2015).

[28] Mocnik, F.-B. *A scale-invariant spatial graph model*. Ph.D. thesis, Vienna University of Technology (2015).

[29] Geisberger, R., Sanders, P., Schultes, D. & Delling, D. Contraction hierarchies: faster and simpler hierarchical routing in road networks. *Proc. 7th Int. Conf. on Exp. Algorithms (WEA)*, 319–333 (2008).

[30] Sporns O, Tonoi G, Kӧtter R (2005). The human connectome: A structural description of the human brain. PLoS Comput. Biol..

[31] van den Heuvel MP, Kahn RS, Goñi J, Sporns O (2012). High-cost, high-capacity backbone for global brain communication. Proc. Natl. Acad. Sci. United States Am..

[32] Bullmore E, Sporns O (2009). Complex brain networks: Graph theoretical analysis of structural and functional systems. Nat. Rev. Neurosci..

[33] Jeong H, Tombor B, Albert R, Oltvai ZN, Barábasi A-L (2000). The large-scale organization of metabolic networks. Nat..

[34] Jeong, H., Tombor, B., Albert, R., Oltvai, Z. N. & Barabási, A.-L. Data as used in the paper ‘The large-scale organization of metabolic networks’. http://www3.nd.edu/~networks/resources/metabolic/CE.dat.gz - accessed on April 14, 2016 (2016). 10.1038/35036627 11034217

[35] Erdős P, Rényi A (1959). On random graphs I. Publ. Math. Debrecen.

[36] Gilbert EN (1959). Random graphs. Annals Math. Stat..

[37] Dijkstra EW (1959). A note on two problems in connexion with graphs. Numer. Math..

[38] Hart PE, Nilsson NJ, Raphael B (1968). A formal basis for the heuristic determination of minimum cost paths. IEEE Transactions Syst. Sci. Cybern..

[39] Staudt, C., Sazonovs, A. & Meyerhenke, H.: NetworKit: a tool suite for large-scale complex network analysis. *Netw. Sci.* **4**(4), 508–530 (2016).

[40] van den Heuvel, M. P., Kahn, R. S., Goñi, J. & Sporns, O. Data as used in the paper ‘High-cost, high-capacity backbone for global brain communication’. http://www.dutchconnectomelab.nl/wordpress/wp-content/uploads/GR_Dataset_n2x40.mat (accessed on June 14, 2018) (2018). 10.1073/pnas.1203593109 PMC3396547 22711833

[41] Trafiklab. GTFS Sverige. https://api.trafiklab.se/samtrafiken/gtfs/sweden.zip - accessed on April 14, 2016 (2016).

